# Amniotic fluid embolism causing multiorgan embolisms and reinforces the need for point-of-care ultrasound

**DOI:** 10.5339/qmj.2023.13

**Published:** 2023-07-28

**Authors:** Nissar Shaikh, Muhammad Fras Alhammad, Seema Nahid, Amara Umm E, Ifrah Fatima, Firdous Ummunnisa, Slawa Abu Yaqoub

**Affiliations:** ^1^Department of Anesthesia, ICU and Perioperative Medicine, Hamad Medical Corporation, Doha, Qatar. E-mail: malhammad1@hamad.qa ORCID ID: https://orcid.org/0000-0002-8241-7939; ^2^Apollo Medical College Hyderabad, Telangana, India; ^3^The University of Missouri–Kansas City (UMKC), Missouri, USA; ^4^Dr. Halima Al Tamimi, Obstetrics & Gynaecology Centre, Doha, Qatar; ^5^Women’s Wellness & Research Center, Hamad Medical Corporation, Doha, Qatar

**Keywords:** Amniotic fluid embolism, cardiac arrest, disseminated intravascular coagulopathy, emergency, multiorgan dysfunction, multiple embolisms, POCUS, peripartum, prevention

## Abstract

Introduction: Pregnant patients are at risk of several possible complications during the peripartum period. Amniotic fluid embolism (AFE) is a peripartum complication with high mortality and morbidity. The sudden entry of amniotic fluid into the maternal circulation causes a rapid and dramatic sequence of clinical events called AFE. The reported incidence of AFE after a cesarean section is around 19%, and after a normal delivery, it is 11%. AFE causing multiple embolisms is not reported in the literature, nor is the use of point-of-care ultrasound (POCUS) in the diagnosis of AFE. We report a case of AFE causing pulmonary and ovarian embolisms.

Case: A 34-year-old pregnant lady had an elective lower section cesarean section (LSCS) for transverse lying and placenta previa under combined spinal and epidural anesthesia. She was gravida 3 para 2 and had regular antenatal check-ups, and she presented for her LSCS at 36 weeks of gestation. Immediately after delivery of the fetus, the patient had convulsions, cardiac arrest, and disseminated intravascular coagulopathy (DIC). Immediately, cardiopulmonary resuscitation started, and the team achieved a return of spontaneous circulation (ROSC) in 3 minutes. DIC was corrected with blood and blood products during this maneuver, and POCUS of the inferior vena cava and heart showed multiple small particles floating, thus confirming the diagnosis of AFE in this patient. The patient was transferred to the intensive care unit (ICU), intubated, and ventilated, necessitating a vasopressor infusion. Computed tomographic pulmonary angiography (CTPA) showed pulmonary embolism and ovarian vein embolism, which were managed with heparin infusion. She was hemodynamically stable and weaned from vasopressors, and the ventilator was then extubated on day 13 of ICU admission. She remained awake and in stable condition. The patient was transferred to the ward and subsequently discharged to go home on the 20^th^-day post-delivery.

Conclusion: AFE can be quickly diagnosed using clinical manifestations and POCUS, and it can be managed early for better patient outcomes. POCUS will show multiple smaller and a few larger amniotic fluid emboli in the heart and vena cava. These larger AFE emboli can migrate and cause multiple embolisms, requiring systemic anticoagulation.

## Introduction

Amniotic fluid embolism (AFE) is a rare and life-threatening complication of pregnancy. The incidence varies between countries and is estimated to be 1.9 per 100,000 pregnancies in the United Kingdom and 6.1 per 100,000 pregnancies in Australia.^1^ AFE is diagnosed by the clinical deterioration of the mother immediately after delivery of the fetus, cardiac arrest, hypoxia, disseminated intravascular coagulation (DIC), and recently by the use of echocardiography. AFE occurs mostly during labor (70%), at the time of normal delivery (11%), or during a cesarean section (19%).^2^ Lower-section cesarean section (LSCS), instrumental delivery, placental abnormalities, preeclampsia, and eclampsia are well-known risk factors.^3^ More than half of AFE patients die in the initial few hours of clinical manifestation, and among the survivors, persistent neurological impairment is reported in more than 60% of cases.^4^ Evans et al. reported their case of AFE with cardiac arrest, used a transesophageal echocardiogram, and found an empty left ventricle and right ventricular dilatation but no amniotic fluid particles.^5^ AFE causing multiple embolisms and the importance of point-of-care ultrasound (POCUS) in the diagnosis of AFE are not reported in the literature. We report a case of AFE causing cardiac arrest (DIC). The POCUS examination showed floating amniotic fluid particles, causing multiple thromboembolisms.

The medical research committee (MRC) and institutional review board (IRB) of our hospital gave their approval for the publication (MRC Permission Number: MRC-04-22-821), and the patient provided written consent for publication.

## Case

**Figure 1.**
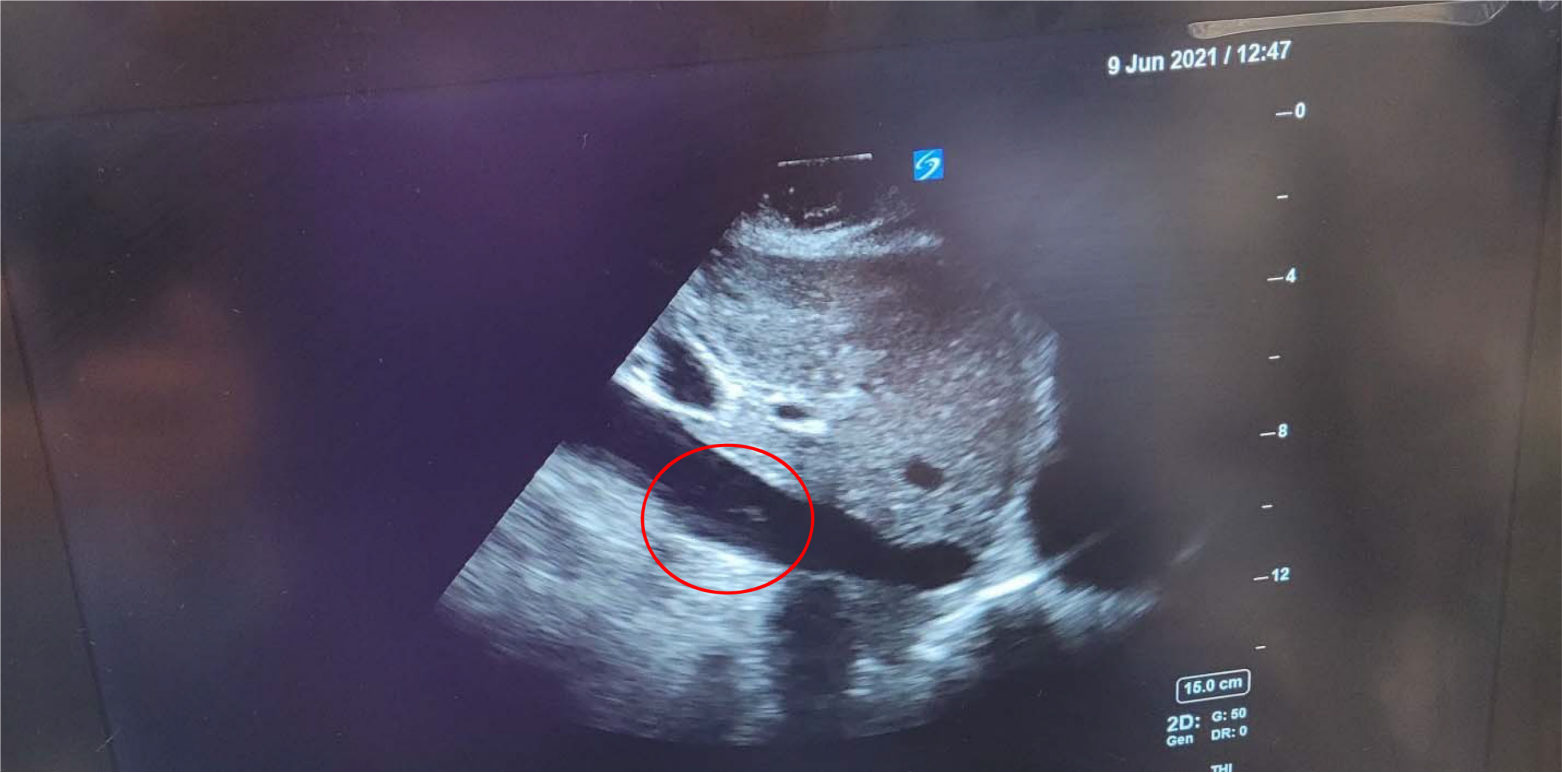
POCUS showing amniotic fluid particles in the IVC.

**Figure 2.**
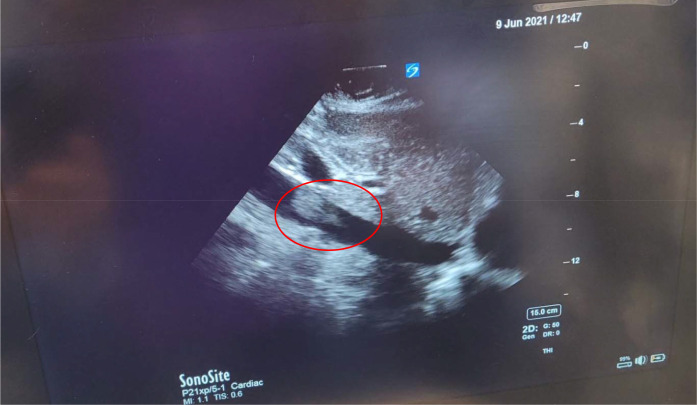
POCUS showing larger particles in the IVC.

**Figure 3.**
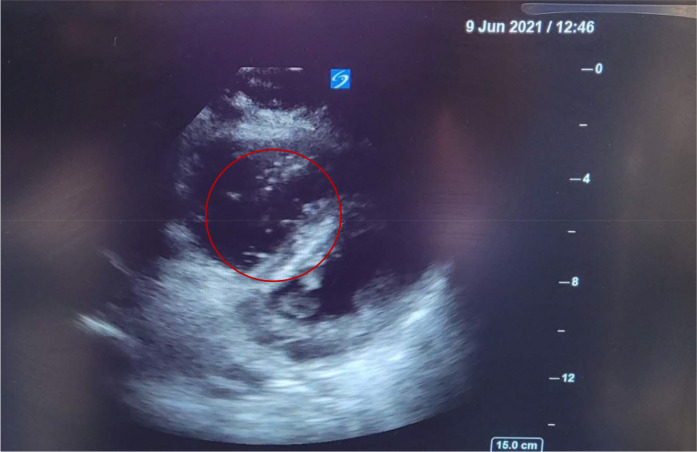
POCUS showing multiple particles on the right side of the heart.

We present the case of a 34-year-old gravida 3, para 2 patient who underwent an elective cesarean section despite having an easy pregnancy. She had a previous caesarean section and was known for having a placenta previa grade 3 with a transverse lie of the fetus. An abdominal ultrasound showed the placenta was posterior, with major previa covering the OS of the cervix. A magnetic resonance imaging (MRI) study on admission showed a posterior placenta previa type III and engorged posterior uterine wall vessels, suggesting underlying uterine wall varicosities. The patient did not have any other comorbidities. The patient was regularly followed in antenatal clinics and was started on dexamethasone for fetal lung maturity. There were no major events during the antenatal period, and she did not have an antepartum hemorrhage. The patient had been scheduled for elective LSCS. She received a combined spinal epidural uneventfully. The surgery proceeded until the fetus was delivered, then suddenly, the patient became unresponsive with no peripheral pulse and in asystole. According to the acute cardiac life support (ACLS), protocol, cardiopulmonary resuscitation (CPR) was started immediately. Adrenaline, calcium gluconate, and sodium bicarbonate were administered along with high-quality CPR, and the return of spontaneous circulation (ROSC) was restored in 3 minutes. The patient was successfully intubated, ventilated, and revived. The ROTEM^®^ study showed profound DIC with a straight line, and details of coagulation and DIC parameters are described in Table 1. The patient was administered blood, blood products, and fibrinogen. POCUS showed several AFE emboli in the inferior vena cava (IVC), a few bigger and multiple smaller amniotic fluid particles (Figures 1 and 2), and a dilated right ventricle (RV) with multiple amniotic fluid particles (Figure 3). In combination with the clinical picture, POCUS, hypoxia, and the DIC pattern, these are strongly suggestive of an AFE as the diagnosis. The patient was transferred to the intensive care unit (ICU) for postoperative monitoring and stabilization. The fetus was pink and cried well, with an Apgar score of 10.

**Figure 4.**
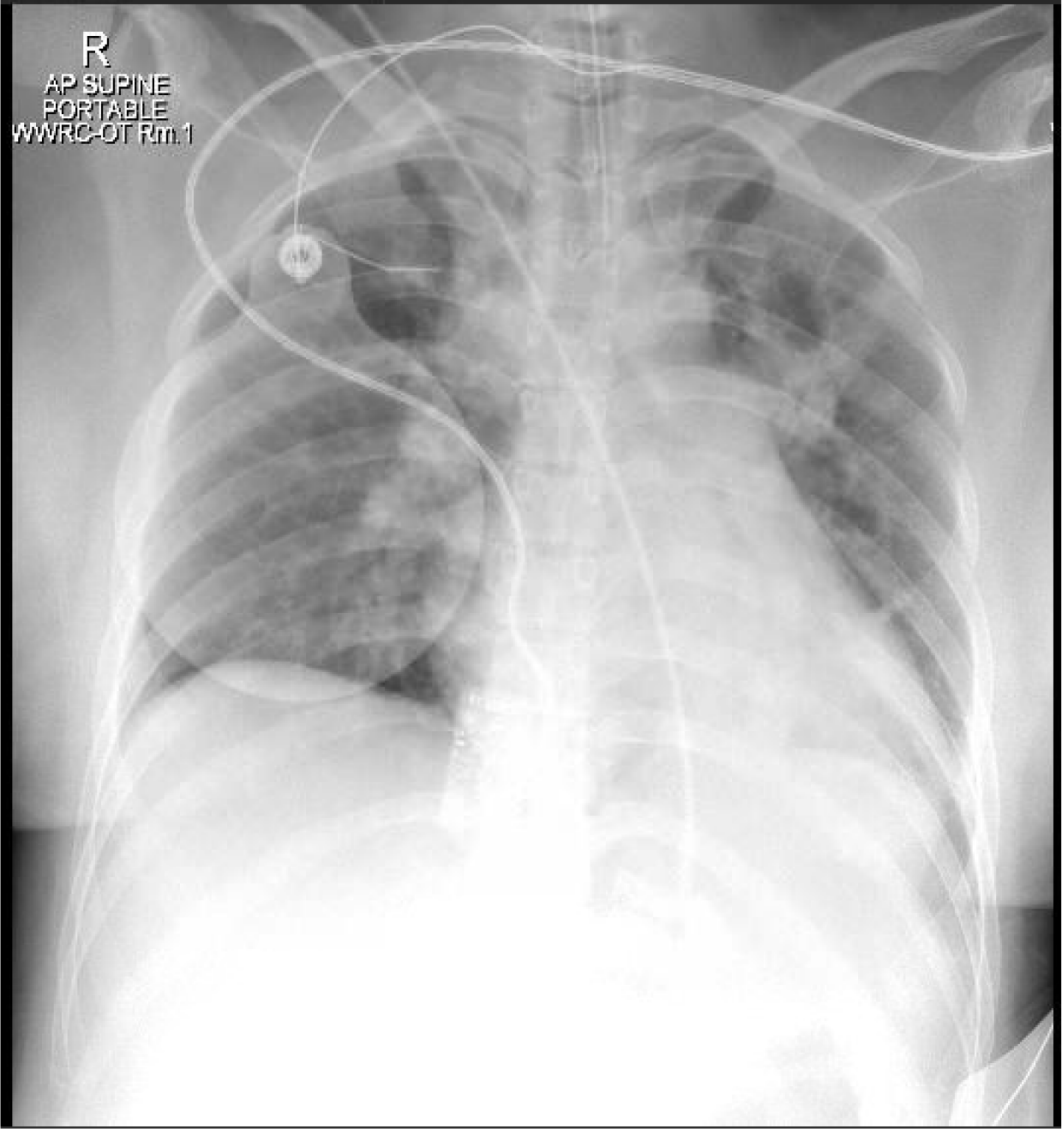
Patient’s chest X-ray showing pulmonary congestion post-cardiac arrest.

**Figure 5.**
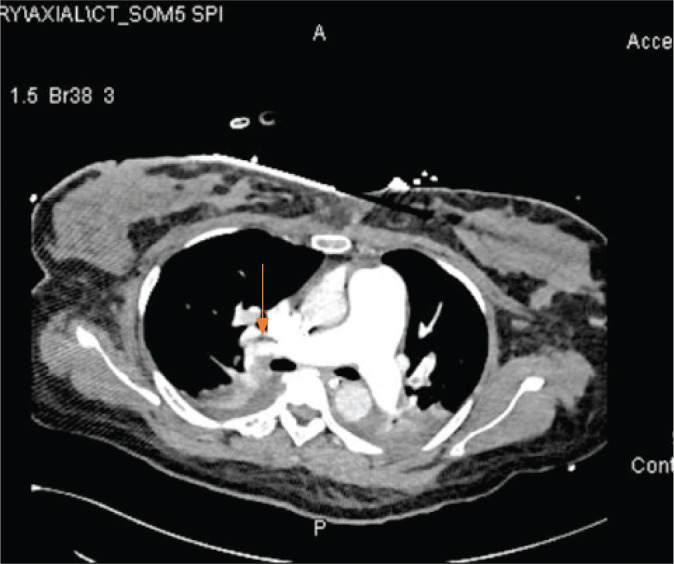
CT chest showing pulmonary embolism.

**Figure 6.**
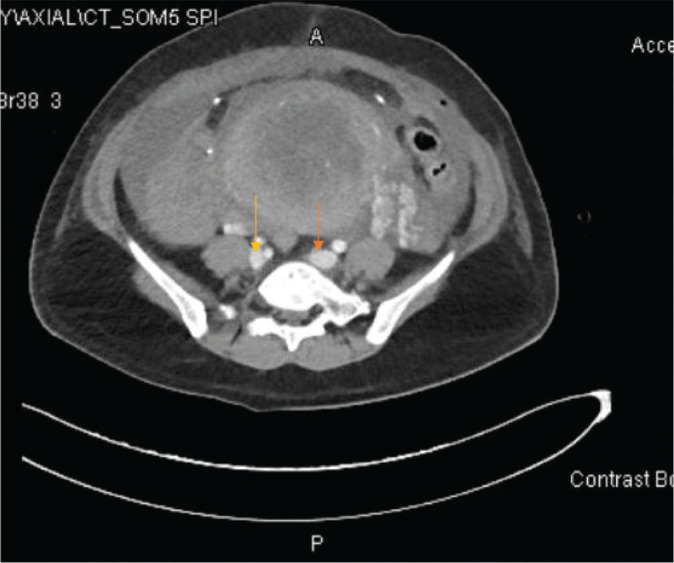
CT abdomen showing bilateral ovarian vein thrombosis.

An urgent bedside echocardiogram performed in the ICU showed normal global systolic left ventricle function, with an ejection fraction of 55% and no regional wall motion abnormality, a severely dilated RV, moderately reduced RV function, and interventricular septal motion that was suggestive of RV pressure overload. The pulmonary artery pressure was mildly increased, and the right atrium was dilated. In the ICU, the patient’s vital signs were not stable, with tachycardia (135 beats/minute) and a blood pressure of 100/60 mmHg (millimeters of mercury) with noradrenaline support. The oxygen saturation (SpO2) was 92%–94%. The patient remained intubated, ventilated, and sedated; a chest x-ray revealed remarkable hepatic congestion with an increased cardiac index (Figure 4). During his ICU stay, the patient developed acute kidney injury after cardiac arrest, requiring three hemodialysis sessions. Computed tomographic pulmonary angiography (CTPA) showed pulmonary embolism and bilateral ovarian (mostly due to embolization of larger amniotic fluid particles seen on POCUS initially) vein embolism on day 3 (Figures 5 and 6), requiring heparin anticoagulation and supportive care. By day 6, she was awake and off vasopressors. On the seventh ICU day, the patient was extubated successfully without any neurological consequences. The patient was transferred to the ward after spending 13 days in the ICU. She had an uneventful stay in the hospital and was discharged on the 20th day.

## Discussion

AFE is a rare, life-threatening clinical condition in the peripartum period. It is suspected when maternal cardiac arrest, seizures, and severe hypoxia followed by DIC occur within 30 minutes of delivery. Getting all these clinical signs and symptoms in all patients is always difficult.^6,7^ There are reports of echocardiograms showing thrombus in the heart and heart chamber changes in patients with AFE.^8^ The use of POCUS in the diagnosis of AFE is not reported in the literature. On POCUS of the IVC and heart, we could see a number of various-sized amniotic fluid particles floating in the IVC and the heart in our patient in addition to the typical signs and symptoms of AFE, namely, cardiac arrest after delivery of the fetus, hypoxia, convulsions, and DIC. Larger ones may have caused pulmonary and ovarian venous embolisms. These visualizations of particles and embolisms are not mentioned in the literature.

From the AFE literature, it is found that the significant pathological findings of AFE on autopsy were pulmonary edema (70%), alveolar hemorrhage, and rarely pulmonary embolism with an amniotic fluid material.^9^

AFE is reported in a range between 1 in 8,000 and 1 in 80,000 deliveries. The reported risk factors for AFE range from trauma to multiple factors. The pathogenesis of AFE is not well understood; however, it is hypothesized that amniotic fluid entering maternal circulation contains fetal cells and antigenic components that will lead to an improper immune response and the release of procoagulant and/or vasoactive substances.^3^ Only 19% of AFEs happen during LSCS, which resembles our case. The amniotic fluid in the maternal circulation stimulates various inflammatory, hormonal, and immunological responses leading to an anaphylactoid reaction; hence, AFE is also termed anaphylactoid syndrome of pregnancy.^10^ New data clearly exclude the role of mechanical obstruction by amniotic fluid debris in increasing pulmonary hypertension and causing right ventricle failure. However, the large debris that was initially detected by POCUS in the IVC and right heart can migrate and get lodged in major veins, causing embolization.^3^

The combination of clinical findings and the POCUS discovery of amniotic particles floating in the circulation may be helpful in the confirmation and early identification of AFE, even though it is still only a clinical diagnostic. POCUS is advanced diagnostic ultrasonography, performed in real-time and at the bedside. POCUS is now utilized in nearly all medical specialties to help with early diagnosis and to initiate therapeutic measures earlier, improving patient outcomes.^11^ At the same time, these patients do not need to be shifted to an ultrasound room, and there is no need for a second physician to perform the imaging. POCUS is successfully used to diagnose pulmonary edema, pleural effusion, cardiac functions, abdominal aortic aneurysms, and deep venous thromboembolism with much higher sensitivity and specificity (more than 90%) compared to traditional diagnostic tests or imaging studies.^11^

The clinical manifestation occurs in most of the patients immediately after delivery of the fetus, as in our case, namely, cardiac arrest (in 100% of patients), hypoxia, cyanosis, coagulopathy (in more than 80% of patients), and seizures, which are reported in up to 15% of patients with AFE.^9^ The diagnosis of AFE is made after ruling out other possible causes. The proposed diagnostic criteria are the following (all of these criteria must exist): (1) The patient has sudden cardiorespiratory collapse or hypotension (systolic blood pressure less than 90 mmHg) with evidence of respiratory distress. (2) The patient has clear DIC. (3) Onset of clinical symptoms during labor or within 30 minutes of delivery. (4) Absence of fever during labor.^3^

Management of AFE remains supportive, taking care of the airway, breathing, and circulation, managing DIC by blood and blood products, and administering fibrinogen. Steroids are frequently used. These patients may require extracorporeal membrane oxygenation (ECMO) support^12^ and peripartum hysterectomy.^13,14^ A multidisciplinary team of anesthesiologists, obstetricians, gynecologists, and intensivists is essential for a patient to have an improved outcome.^10^ In our patient, the follow-up CTPA for hypoxia showed pulmonary and ovarian vein embolization of larger particles of AFE visualized initially by POCUS, requiring anticoagulation after the DIC was corrected. As mentioned, AFE is a life-threatening condition, with the older literature reporting an 86% mortality rate, whereas the newer reports refer to a 30% mortality of around 30%.^9,15^

## Conclusion

AFE is a rare, life-threatening condition in the peripartum phase. We report this case of AFE that occurred immediately after the delivery of the fetus by LSCS, with the patient going into cardiac arrest. The situation was rapidly reversed, and the patient was eventually discharged home after 19 days without any neurological consequences.

Several small and a few larger floating particles in the IVC and heart, along with the clinical signs and symptoms, can corroborate the presence of AFE during a POCUS examination. Hence, one should perform POCUS early during aggressive resuscitation with blood and blood products. The amniotic fluid’s larger particles can cause multiple embolisms, leading to multiorgan dysfunction requiring systemic anticoagulation.

### Conflict of interest statement

All authors declare that they do not have any conflict of interest either academic or financial.

### Authors contributions statement

Nissar Shaikh: written the manuscript.

Muhammad Firas Alhammad: Collected and assembled patient history, investigations, manuscript review.

Seema Nahid: written the manuscript

Amara Umm E: manuscript review.

Ifrah Fatima: manuscript review.

Firdous Ummunnisa: manuscript review.

Slawa Abu Yaqoub: manuscript review

## Figures and Tables

**Table 1.** Showing DIC profiles.

**Day**	**PT (seconds)**	**apTT (seconds)**	**INR**	**Fibrinogen (g/L)**	**d-Dimer (mg/L)**	**Platelets (×10^3^/μL)**
During event	43.1	134.2	4.0	0.11	>35	50
During resuscitation	37.7	126.5	3.7	0.43	35.2	56
Post resuscitation	15.3	1.5	1.5	1.8	15.2	62
ICU day 1	14.1	41.7	1.4	2.6	18.2	46
ICU day 2	11.1	36.6	1.0	3.3	10.7	73

DIC: disseminated intravascular coagulation; PT: prothrombin time; apTT: activated partial thromboplastin time; INR: international normalizing ratio; ICU: intensive care unit.
